# Flap Salvage Following Postoperative Venous Thrombosis Diagnosed by Blood Glucose Measurement in the Flaps

**Published:** 2011-06-22

**Authors:** Hisako Hara, Makoto Mihara, Mitsunaga Narushima, Takumi Yamamoto, Takeshi Todokoro, Jun Araki, Takuya Iida, Isao Koshima, Timothy Weng Hoh Shim

**Affiliations:** Department of Plastic and Reconstructive Surgery, The University of Tokyo, Japan

## Abstract

**Objective:** This is the first paper to report the measurement of blood glucose in flaps to detect early flap congestion and flap salvage following immediate flap exploration. **Methods:** We performed blood glucose measurement in a superficial circumflex iliac artery perforator flap and an anterolateral thigh flap postoperatively using Medisafe-Mini (Terumo, Japan), a regular capillary blood glucose–monitoring device. **Results:** The blood glucose measurements were low when in early venous thrombosis. Low capillary blood glucose levels were detected early, even before flap discoloration occurred. **Conclusions:** Low capillary blood glucose level in congested flaps is an early sign of venous thrombosis. The incorporation of blood glucose measurement in addition to clinical monitoring aids in early detection and possible reduction postoperative complications due to venous thrombosis.

The incidence of thrombosis at the anastomotic site following free flaps is 3% to 10%^1^,^2^ and is an ever-present risk irrespective of how experienced the surgeon is. Although flap congestion may be more damaging than ischemia, it is sometimes difficult to diagnose early venous thrombosis, in contrary to the diagnosis of arterial insufficiency. Early detection of anastomotic site thrombosis and rapid salvage operation is essential for flap survival, so establishing a reliable method for flap monitoring is crucial. This articles reports cases in which blood glucose measurement (BGM) aided early detection and flap salvage, highlighting its usefulness in early diagnosis of flap congestion.

## METHODS

**Figure 1:**
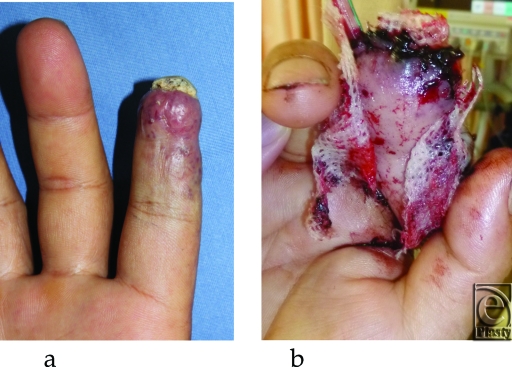

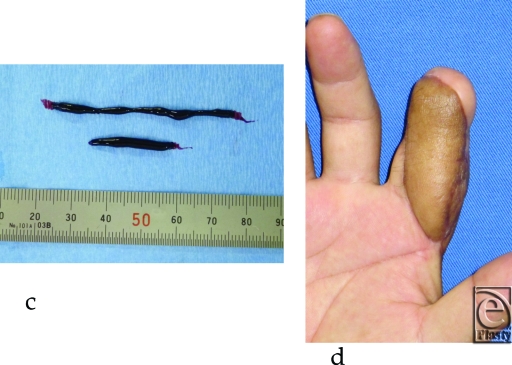
Case 1: (a) Preoperative image of case 1. (b) Image on postoperative day 1. (c) Venous thrombus found in the anastomosis site. (d) Image of postoperative day 70. The flap survived totally.

Case 1 was a 47-year-old landscape gardener, who had cut his right index finger with a saw 6 years ago. Although the wound healed without any treatment, his finger became sore 1 year after the injury because of an arterovenous fistula. The pain intensified 5 years after the injury with necrosis in his fingertip (Fig 1a). A radiograph showed bone absorption of the distal phalanx.

Case 2 was a 32-year-old man, whose right hand was crushed by a stamping machine causing avulsion amputation injury to all his right fingers. He underwent many operations including an abdominal flap, groin flap, and skin graft transplantation in another hospital. We subsequently performed a great toe transplantation to his right thumb 1 year ago.

The flaps were monitored by pinprick test and BGM using Medisafe-Mini (Terumo, Japan), which is usually used by diabetic patients. Blood glucose measurement was performed immediately after the operation, upon the patients' arrival in the ward, 1 hour after returning to the ward, the next morning and arbitrarily thereafter. In addition, blood glucose was measured when anastomotic site thrombosis was suspected.

## RESULTS

In case 1, resection of the arterovenous fistula and reconstruction of the right index fingertip and pulp with a hemipulp flap and superficial circumflex iliac artery perforator (SCIP) flap was performed. The operation was uneventful, and the color of the flaps were good immediately after the operation (Fig 1b). The color of the distal tip of the SCIP flap turned purple the next morning, but the color of the most part of the flap remained good (Fig 1c). A reoperation was performed because the blood glucose level in the SCIP flap was low (<20 mg/dL), while that of the hemipulp flap was 146 mg/dL. A 5-cm venous thrombus was found at the site of anastomosis (Fig 1d). Thrombectomy and venous reanastomosis was performed. The entire flap survived 70 days after the operation, (Fig 1e).

**Figure 2:**
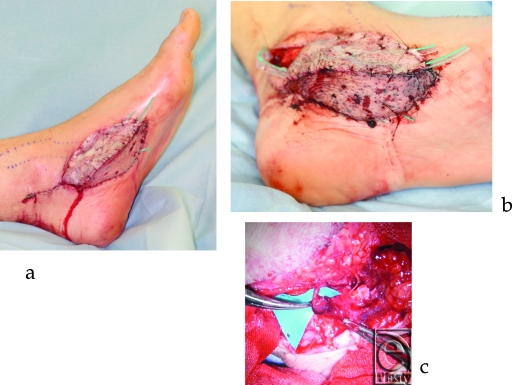
Case 2: (a) Postoperative image of case 2. (b) Three hours after the patient returned to his room. (c) Venous thrombus found in the anastomosis site.

In case 2, a left medial plantar flap was transplanted to the right index finger pulp and a left anterolateral thigh (ALT) flap was transplanted to the medial plantar flap donor site. The flap color was good and the blood glucose level in the ALT flap was 90 mg/dL when the patient returned to the ward (Fig 2a). The flap turned partially purple after 3 hours, and venous thrombosis was suspected (Fig 2b). We decided to explore the flap because the flap blood glucose level dropped to 36 mg/dL. Indeed venous thrombosis occurred, thrombectomy and venous reanastamosis was performed (Fig 2c). The flap blood glucose level normalized to around 100 mg/dL after the reoperation, and the flap completely survived.

## DISCUSSION

This is the first paper to report the utilization of flap blood glucose level monitoring to detect early congestion of the flaps prior to color change of the skin paddle, and a guide in decision making for flap exploration. Studies using porcine intestine, porcine skin flap, or human skin flap showed that blood glucose levels are reduced in congested and ischemic conditions.^3^^-^5 In addition, parts of the flap where blood glucose level is low will undergo necrosis.^6^

**Figure 3:**
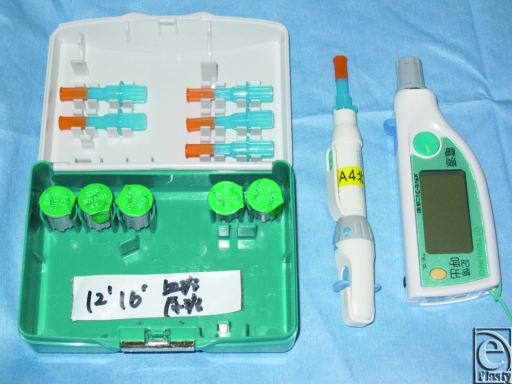
Medisafe-Mini (Terumo, Japan).

We monitored the flaps by measuring blood glucose levels using Medisafe-Mini (Fig 3), which is routinely used for regular capillary blood glucose measurements. The procedure is rapid and simple and requires only minimal amounts of blood (10–20 µL). Furthermore, this method is more quantitative than the traditional ways of flap monitoring, such as observing flap color, flap turgor, or the pinprick test. It provides a surrogate reflection of the adequacy of flap perfusion and allows objective comparisons between tests. On the contrary, the needle prick test only yields subjective information on flap perfusion.

The frequency of flap monitoring differs greatly among different microsurgery units. We performed BGM immediately after the operation, upon the patients' arrival in the ward, 1 hour after returning to the ward, the next morning and arbitrarily thereafter. Blood glucose measurement may be incorporated as an adjunct during routine clinical monitoring at specified intervals. Although continuous blood glucose monitoring is probably more useful to detect venous thrombus earlier, it requires specialized instruments and is not practical.

The mechanism of reduction in blood glucose levels in congested flaps has not been elucidated. Sakakibara et al^5^ hypothesized that in venous thrombosis, flap blood glucose concentration is reduced due to continual uptake of glucose from the stagnated blood for tissue metabolism. Several studies showed that blood glucose is also reduced with ischemia, and that this reduction is more rapid and extensive than that associated with congestion.^3^,^4^ In addition, the concentration of lactate rises more rapidly and extensively with ischemia than with congestion. These findings suggest that the mechanism of reduced blood glucose in congestion and ischemia can be attributed to both a deficiency in glucose supply as well as increased anaerobic respiration in the tissues.^6^

The cutoff value, sensitivity, and specificity of BGM in diagnosis of early flap congestion have not been determined and awaits to be elucidated by further research. An integration of monitoring methods such as BGM, conventional clinical monitoring and other adjuncts may aid in early and rapid diagnosis of postoperative venous thrombosis.
